# Studies in Relation to the Production of Pain by the Weather

**Published:** 1879-03

**Authors:** J. T. Everett

**Affiliations:** Sterling, Ills.


### ARTICLE III.

### STUDIES IN RELATION TO THE PRODUCTION OF PAIN BY WEATHER. By J. T. EVERETT, A.M., M.D., Sterling, Ills.

It has long been a popular belief that parts which are the seat of old traumatisms, chronic rheumatism and old tissue deposits become the seat of pain upon the approach of storms. This no one has attempted to explain. Until quite recently I have seen no other consideration of the subject in our literature than an admission of the general fact, without suggestion as to its cause and character.

As I had been in early life the subject of coxalgia, and since then have been a periodical sufferer from the same trouble, I commenced, in the spring of 1866, a series of observations, with a view to solving the problem, if possible.

Careful observations, extending over this period of 12½ years, have established, in my mind, the main fact, but have failed to give me an explanation of the phenomena here considered.

In July, 1878, I addressed the following circular to two hundred and fifty of the most prominent medical men in this country and Europe:

STERLING, ILLS., July 1, 1878.

DEAR DOCTOR: Will you have the kindness to fill out this blank, and oblige,

Yours resp'y, J. T. EVERETT.

Etiology of Chronic Rheumatism.
Pathology of same.
Atmospheric Influence.
Ozonic Influence.
Electrical Influence.
Influence of Humidity.
Relation of Barometric Pressure.
Relation of Storm Waves.

To this circular the greater number very kindly replied at some length, giving many valuable hints and facts bearing upon the subject.

The majority believed that either electricity or magnetism was the principal factor, but did not know in what manner the cause operated.

A few suggested that ozone might be the primary factor; but they too failed to explain how or why it produced pain. But very few claimed that there was any direct relation between barometric pressure and the physical symptoms; while all admitted the close connection between storm waves and the advent of pain.

Dr. S. Weir Mitchell, in his very able and instructive paper upon "the relation of pain to weather," says:—"the separate factors of storms, such as lessened pressure, rising temperature, greater humidity and winds, appear, as a rule, to be incompetent, when acting singly, to give rise to attacks of pain. Either, then, it is the combination which works the mischief, or else there is, in times of storms, some as yet unknown agency productive of evil. Such an agent may be either electricity or magnetism."

These statements seem to express the general opinion of the profession at large upon the subject.

Dr. Mitchell found that rheumatic subjects were too sensitive for careful observation. To me, however, they have proved most useful; perhaps because a thirty years experience with this intangible element has made me rather more expert in eliciting the true symptoms of other sufferers, and in rejecting those that are false.

I have observed and tabulated 14 cases of chronic rheumatism, 2 of traumatic neuralgia, 24 of chronic metritis and 12 of malignant anæmia of specific origin.

Many other patients had such negative symptoms as were useless in establishing results ; they are here omitted.

It has lately become possible to ascertain the proximity of storm belts through the reports of the U. S. Signal Service office; and the observations made by me prior to the year 1871 are here omitted in consequence of defects due to the absence of these meteorological observations.

Since that year the occurrence of increased pain can be seen to have direct connection with the supervention of either rains, snows or dry storms.

I find that there is a close relation between the occurrence of pain and lessened pressure. The lessened pressure *per se* probably does not cause the pain ; rather is it true that a common factor produces both. That humidity is still more closely allied to the occurrence of pain is also true. Yet this alone does not seem sufficient to either produce or relieve the symptom. Humidity, however, in connection with other storm influences, seems to be potent in relieving pain. Does this humidity furnish a better conductor for the passage of the systemic electricity from the body, or does it lessen the amount of magnetic induction ? Whether the pain felt depends upon excess or deficiency of magnetism or excess or deficiency of electricity, is a question *sub judice*.

We know that the electric current is at times capable of allaying pain, but why? Temperature has little or no influence, either one way or another.

As to the time of day, there is a preponderance of evidence in favor of a period of greatest suffering between 9 and 11 p.m., and of a period of great but less distress between 8 and 10 a.m. These results differ materially from those obtained by Dr. Mitchell.

The longest interval between the onset of pain and the appearance of the storm, was in my own case, the pain appearing at 11:30 a.m., March 28, and no storm reaching my vicinity until 4:45 p.m., March 30th.

Upon the arrival of either rain, snow or a dry storm, the pain soon subsides.

My observations teach that Dr. Mitchell is correct in assuming that: "the amount of pain bears no relation to the amount of rainfall; neither does the abruptness of the barometric depression, the length, direction or severity of the storm." But that the location in the center or at the edges of the storm belt has a marked effect upon the degree and persistence of the pain, I am fully convinced. The nearer the center of the belt, and the more abrupt the invasion, the more intense is the pain and the shorter the duration; while at the edges of the belt, less fierce is the attack and more slow the retreat.

Thus it would appear that the morbific influence is most intensely active in advance of the storm, for a distance of 200 or 300 miles, according to the rapidity with which the storm center moves. If the velocity be great, the *influence* for pain is proportionately intense; if the storm moves slowly, this *influence* is less distinct and is felt proportionately at an earlier period. Upon the edges of the storm this influence gradually decreases and finally disappears. This influence would thus seem to be governed by the laws which control matter, or the forces which act through matter.

It would seem also to be governed by the laws which control the accumulation of frictional electricity in front of the rubber, the clouds acting the part of this rubber upon the negative earth. Yet this is not a true analogy, since, in the electrical machine, the rubber must be perfectly dry, with a dry conducting medium, while in the case of the earth the rubber is a mass of aqueous vapor, and the cylinder more or less moist. Then, too, in the terrestrial machine the active agent collects and accumulates in front, while in the artificial apparatus this occurs at the points of the conductor.

The nervous system of the average adult, in its normal condition, is quite uniform in the amount of electricity generated and the amount of heat produced by tissue metamorphosis. In disease, however, the amount of heat generated may vary by 6 or 7 degrees, while the electricity may vary from the normal 9 or 10 times. Any disease which tends to check tissue changes, transpiration, assimilation or to favor tissue deposit, or to retard the play of the vital affinities, predisposes to this condition of the system.

The individual who readily "takes cold" has rheumatic pains. He who works hard and greatly fatigues any particular set of muscles, experiences there the same kind of pain. In chronic inflammation, where deposit has taken place ; in the limbs of the inebriate where alcohol has anæsthetised and lessened the tissual change ; in cases of pernicious anæmia or blood poisoning from any cause ; in the professional man who has fatigued the brain by laborious and exhausting study, thereby retarding nutrition and assimilation ; in the nervous woman who has suffered from exhausting influences ; in the debauchee, and, in short, in every case where the laws of life are violated, this condition supervenes.

It is a noticeable fact that, aside from storm influences, terrestrial magnetism exerts a wonderful influence upon this class of patients.

In 1867–8 and also in 1872, when the Auroræ were of exceptional brilliancy and frequency, the periods of pain were more frequent, more severe and of longer duration, in proportion to the storm influences.

During the prevalence of the Auroræ, the pain was more intense, and upon their disappearance it also subsided. When the sun spots are more numerous, during an eclipse and also during the conjunction of the heavenly bodies, the pain is more severe, and gradually ceases as these phenomena disappear.

In 14 cases of chronic rheumatism, extending over a period of seven years (a total number of 168 months), the number of false pain indications was 39 ; while the false barometric indications were nine in a record of 1,935 storms. In two cases of traumatic neuralgia recorded for one year, each over a period of 24 months, the false pain indications were two, with no barometric failures in 300 storms.

In 24 cases of chronic metritis, extending over a period of 258½ months, there occurred 3,090 storms and only 68 false pain and 11 false barometric indications.

In 12 cases of malignant anæmia, extending over a period of 133 months, there were 13 false pain indications and three barometric fallacies in 1,558 storms.

In the following table the first column gives the number of the case; the 2nd, the number of months; and the 3d, the year in which the cases were observed. The 4th gives the number of storms; the 5th, the pain indications; the 6th, the barometric indications of storms; the 7th, the number of rain storms; the 8th; the number of snow storms; the 9th, the number of cloudy days; the 10th, the number of false pains; and the 11th, the number of false barometer indications of storms; while the 12th and 13th give the approximate percentage of errors both of pain and barometer.

In this table I have included under the head of "storms" every slight sprinkling of rain or snowfall or appearance of clouds, as well as the most disastrous floods and tornadoes.

| No. of Case. | No. of Months. | Year. | No. of Storms. | No. of Pain Indications. | No. of Barometric Indications. | No. of Rains. | No. of Snows. | No. of Cloudy. | False Pain Indications. | False Barometric Indications. | Per cent. of False Pain Indications. | Per cent. of False Barometric Indications. |
|---|---|---|---|---|---|---|---|---|---|---|---|---|
| R 1 | 12 | 1870–1 | 140 | 149 | 144 | 56 | 21 | 63 | 9 | 4 | .060 | .030 |
| R 2 | 12 | 1872–3 | 128 | 128 | 127 | 52 | 31 | 45 | 0 | 1 | .000 | .000 |
| R 3 | 12 | 1872–3 | 128 | 130 | 127 | 52 | 31 | 45 | 2 | 1 | .014 | .007 |
| M 17 | 12 | 1872–3 | 128 | 127 | 127 | 52 | 31 | 45 | 1 | 1 | .007 | .007 |
| M 18 | 12 | 1872–3 | 128 | 130 | 127 | 52 | 31 | 45 | 2 | 1 | .014 | .007 |
| M 19 | 16 | 1873 | 60 | 61 | 60 | 31 | 2 | 27 | 1 | 0 | .014 | .000 |
| M 15 | 6 | 1873 | 60 | 60 | 60 | 31 | 2 | 27 | 0 | 0 | .000 | .000 |
| A 41 | 12 | 1873–4 | 138 | 140 | 138 | 101 | 21 | 16 | 2 | 0 | .014 | .000 |
| A 42 | 12 | 1873–4 | 138 | 137 | 138 | 101 | 21 | 16 | 1 | 0 | .007 | .000 |
| A 43 | 12 | 1873–4 | 138 | 141 | 138 | 101 | 21 | 16 | 3 | 0 | .022 | .000 |
| R 4 | 12 | 1873–4 | 138 | 138 | 138 | 101 | 21 | 16 | 0 | 0 | .000 | .000 |
| R 5 | 12 | 1873–4 | 138 | 140 | 138 | 101 | 21 | 16 | 2 | 0 | .014 | .000 |
| M 26 | 12 | 1873–4 | 138 | 140 | 138 | 101 | 21 | 16 | 2 | 0 | .014 | .000 |
| M 27 | 12 | 1873–4 | 138 | 142 | 138 | 101 | 21 | 16 | 4 | 0 | .028 | .000 |
| R 6 | 12 | 1874–5 | 141 | 141 | 142 | 88 | 23 | 30 | 0 | 1 | .006 | .007 |
| R 7 | 12 | 1874–5 | 141 | 139 | 142 | 88 | 23 | 30 | 2 | 1 | .014 | .007 |
| R 8 | 12 | 1874–5 | 141 | 150 | 142 | 88 | 23 | 30 | 9 | 1 | .063 | .007 |
| M 20 | 12 | 1874–5 | 141 | 151 | 142 | 88 | 23 | 30 | 10 | 1 | .065 | .007 |
| M 21 | 12 | 1874–5 | 141 | 128 | 142 | 88 | 23 | 30 | 13 | 1 | .092 | .007 |
| M 22 | 12 | 1874–5 | 141 | 150 | 142 | 88 | 23 | 30 | 9 | 1 | .063 | .007 |
| M 23 | 12 | 1875–6 | 152 | 149 | 151 | 79 | 30 | 48 | 3 | 1 | .022 | .007 |
| M 24 | 12 | 1875–6 | 152 | 149 | 151 | 79 | 30 | 43 | 3 | 1 | .022 | .007 |
| M 25 | 12 | 1875–6 | 152 | 139 | 151 | 79 | 30 | 43 | 13 | 1 | .092 | .007 |
| M 28 | 12 | 1875–6 | 152 | 150 | 151 | 79 | 30 | 43 | 2 | 1 | .014 | .007 |
| M 29 | 12 | 1875–6 | 152 | 151 | 151 | 79 | 30 | 43 | 1 | 1 | .007 | .007 |
| A 44 | 12 | 1875–6 | 152 | 149 | 151 | 79 | 30 | 43 | 3 | 1 | .022 | .007 |
| A 45 | 12 | 1875–6 | 152 | 151 | 151 | 79 | 30 | 43 | 1 | 1 | .007 | .007 |
| A 46 | 12 | 1875–6 | 152 | 151 | 151 | 79 | 30 | 43 | 1 | 1 | .007 | .007 |
| R 9 | 12 | 1876–7 | 140 | 141 | 140 | 100 | 30 | 10 | 1 | 0 | .007 | .000 |
| R 10 | 12 | 1876–7 | 140 | 142 | 140 | 100 | 30 | 10 | 2 | 0 | .014 | .000 |
| R 11 | 12 | 1876–7 | 140 | 141 | 140 | 100 | 30 | 10 | 1 | 0 | .007 | .000 |
| R 12 | 12 | 1876–7 | 140 | 140 | 140 | 100 | 30 | 10 | 0 | 0 | .000 | .000 |
| R 13 | 12 | 1876–7 | 140 | 139 | 140 | 100 | 30 | 10 | 1 | 0 | .007 | .000 |
| R 14 | 12 | 1876–7 | 140 | 150 | 140 | 100 | 30 | 10 | 10 | 0 | .065 | .000 |
| M 30 | 10 | 1876–7 | 120 | 120 | 120 | 80 | 30 | 10 | 0 | 0 | .000 | .000 |
| M 31 | 9 | 1876–7 | 118 | 119 | 118 | 78 | 30 | 10 | 1 | 0 | .007 | .000 |
| M 32 | 9 | 1876–7 | 118 | 119 | 118 | 78 | 30 | 10 | 1 | 0 | .007 | .000 |
| M 33 | 11 | 1876–7 | 130 | 130 | 130 | 88 | 32 | 10 | 0 | 0 | .000 | .000 |
| M 34 | 11 | 1876–7 | 130 | 130 | 130 | 88 | 32 | 10 | 0 | 0 | .000 | .000 |
| M 35 | 11 | 1876–7 | 130 | 130 | 130 | 88 | 32 | 10 | 0 | 0 | .000 | .000 |
| M 36 | 12 | 1876–7 | 140 | 141 | 140 | 100 | 30 | 10 | 1 | 0 | .007 | .000 |
| M 37 | 11.5 | 1877–8 | 148 | 147 | 149 | 54 | 21 | 73 | 1 | 1 | .007 | .007 |
| M 38 | 12 | 1877–8 | 150 | 150 | 150 | 54 | 23 | 73 | 0 | 0 | .000 | .000 |
| A 39 | 12 | 1877–8 | 150 | 150 | 150 | 54 | 23 | 73 | 0 | 0 | .000 | .000 |
| A 40 | 12 | 1877–8 | 150 | 150 | 150 | 54 | 23 | 73 | 0 | 0 | .000 | .000 |
| M 16 | 6 | 1878 | 71 | 72 | 73 | 21 | 16 | 34 | 1 | 2 | .007 | .014 |
| A 47 | 6 | 1877 | 79 | 79 | 79 | 33 | 7 | 39 | 0 | 0 | .000 | .000 |
| A 48 | 6 | 1877 | 79 | 79 | 79 | 33 | 7 | 39 | 0 | 0 | .000 | .000 |
| A 49 | 7 | 1877–8 | 80 | 80 | 80 | 33 | 8 | 39 | 0 | 0 | .000 | .000 |
| A 50 | 12 | 1877–8 | 150 | 149 | 150 | 54 | 23 | 73 | 1 | 0 | .007 | .000 |
| T N 51 | 12 | 1877–8 | 150 | 150 | 150 | 54 | 23 | 73 | 1 | 0 | .000 | .000 |
| T N 52 | 12 | 1877–8 | 150 | 150 | 150 | | | | | | | |

It will be seen from the facts here tabulated—the condensed statistics of many observations—that there is a remarkable correspondence between the prevalence of storms and of pain, thus suggesting that the latter springs directly from some occult storm influence. What is that influence, how does it operate and how can it be neutralized?

It certainly does not depend upon any single factor, unless it be electricity or magnetism. Is it not possible that the conductivity of diseased tissues may become so impaired, and that when storms approach and the air is charged to repletion with the electrical fluid, none escapes from the surface of the body? The systemic electricity might thus accumulate in the economy producing pain in indurated parts, in its attempt to pass off to the earth. Thus upon the occurrence of humidity a ready conductor is found, an equilibrium established and the pain subsides.

If this were true, an electrical current might establish an equilibrium, and thus speedily mitigate the pain. This I have attempted many times with questionable results.

Another indication of the fallacy of such a theory is the known fact that, when we insulate a patient suffering with acute inflammatory rheumatism, the pain ceases within six or eight hours.

It may be that in these patients not enough systemic electricity is generated, and that insulation by retarding conduction permits an accumulation of the electrical fluid. Can it be that the magnetic wave passes into the patient by induction, and that, in its attempt to pass out by conduction, the pain arises as in the first hypothesis?

Is it possible that the electricity preceding the storm converts a portion of the systemic oxygen into ozone, and that, this being an electro-negative oxygen, the absorption of another equivalent of electricity may cause pain in its passage from one tissue to another?

Although the coincidence of ozone and pain must be less frequent than many other single factors of storms, may it not be, that this ozone being a stimulant, increases nerve metamorphosis, and increases the quantity of systemic electricity thus secondarily causing pain.

Which of these theories, if any, may be capable of demonstration, and, in default of all these, what may be the true explanation of the coincidence of pain and storms, is the problem I have endeavored to set forth.

